# Long-Term Changes in Macrophyte Distribution and Abundance in a Lowland River

**DOI:** 10.3390/plants11030401

**Published:** 2022-01-31

**Authors:** Andrej Peternel, Alenka Gaberščik, Igor Zelnik, Matej Holcar, Mateja Germ

**Affiliations:** 1 Department of Biology, Biotechnical Faculty, University of Ljubljana, Jamnikarjeva 101, 1000 Ljubljana, Slovenia; andrej.peternel@gmail.com (A.P.); alenka.gaberscik@bf.uni-lj.si (A.G.); matej.holcar@bf.uni-lj.si (M.H.); mateja.germ@bf.uni-lj.si (M.G.); 2 Environmental Agency of the Republic of Slovenia, Vojkova 1b, 1000 Ljubljana, Slovenia

**Keywords:** macrophytes, lowland river, long-term changes, environmental parameters, ecological status, Slovenia

## Abstract

The aim of this study was to reveal the changes of macrophyte community over time and along the course of the Ižica River. In 1996, 2000, and 2016, we surveyed the distribution and abundance of macrophyte species in the lowland Ižica River, which originates in the town of Ig and then flows through an agricultural landscape. We calculated the River Macrophyte Index (RMI), which reflects the ecological status of the river. In 2016, ecomorphological conditions of the river, using the Riparian, Channel and Environmental inventory, were also assessed. In just 10.5 km of the river, we identified 27 taxa of macrophytes, among which *Potamogeton natans*, *Sagittaria sagittifolia*, and *P. perfoliatus* were the most abundant. Detrended correspondence analysis showed that, in 1996, the surveyed stretches differed more according to macrophyte composition than in the following years. The assessed environmental parameters explained 43% of the variability of the macrophyte species; riverbank stability explained 20%, riverbed structure 10%, while vegetation type of the riparian zone and bottom type explained 7 and 5%, respectively. The species composition of the macrophyte community revealed significant changes over the years of the riverine ecosystem. Comparison of RMIs in 1996 revealed better conditions in the upper and middle part of the river, while in 2016, the situation was the opposite, since the conditions in the upper part deteriorated significantly over time, while the lower part of the river had the best ecological status. These changes may be due to a considerable increase in the population of the settlement Ig, while better status in the lower course of the river may be a consequence of improvements in the infrastructure and the use of sustainable agricultural practices in the catchment due to the establishment of a formal area of protection.

## 1. Introduction

Rivers are ecosystems that manifest great dynamics in time and space [1]. Aquatic macrophytes are well adapted to seasonal variations of flow rate and flow velocity [2,3]. Macrophytes and riparian vegetation respond to environmental parameters and internal succession mechanisms across the transverse and longitudinal river dimensions and over time [4]. Macrophytes are involved in energy flow, nutrient cycling, and sedimentation processes, and are essential to the structure and functioning of the river ecosystem [5]. They increase habitat heterogeneity and complexity and affect a variety of organisms such as invertebrates, fish, and water birds [6,7], providing food and refuge. They also affect water quality [8] by uptake of nutrients, particularly those containing phosphorus and nitrogen, both from water and sediment [9]. On one hand, macrophytes contribute to river self-purification process as they store nutrients, but on the other hand, they can exert a significant effect on the eutrophication process, as they release these nutrients during decay [10]. They are especially important in lowland streams, where they may occur in high abundance [11].

Macrophytes show differing sensitivities to various natural and human pressures. These differences in sensitivity make them good indicators of the ecological status of a river [8,12,13], as well as indicators of the presence of different toxic substances in the sediment and the water [6,14,15,16]. The presence and abundance of macrophytes depend directly on water quality, depth, flow, substrate characteristics, and other environmental factors [17]. Their role is especially important in lowland watercourses since they increase the variability of habitats and physical conditions in a river [18]. In watercourses flowing through an agricultural landscape, macrophyte assemblages are well developed since these watercourses usually have poorly developed riparian zones and high input of nutrients [19,20].

The majority of rivers in Europe have been affected to different extents by human activity [21]. Introduction of new standards in river and catchment management, and new legislation, such as the Water Framework Directive (WFD), will reduce these pressures (Directive 2000/60/EC) [22]. In particular, the rivers flowing through agricultural landscapes are often exposed to high influxes of nutrients, as well as morphological alterations, both of which negatively influence the biodiversity of riverine ecosystems [23]. Beside the valuable role of macrophytes as the indicators of the current human pressure, aquatic macrophytes have been used by many researchers to monitor the long-term changes in rivers [24,25,26,27,28,29], as well as in lakes [30,31,32].

In 1996, 2000 and in 2016, we completed surveys to estimate potential changes of the presence, abundance, and distribution of macrophytes in the Ižica River (Slovenia) as it originates in the settled area of the town Ig, and then flows through an agricultural landscape. In addition to the WFD implementation in 2008, a part of the catchment of the Ižica River was protected as a Landscape Park within the same timeframe. On the other hand, the population development index of the town of Ig, which spreads in the narrowest part of the catchment area of the Ižica’s source was, between 2002 and 2012, the highest within the Ljubljana metropolitan region [33]. Thus, we hypothesized that the composition of macrophyte community in the river would therefore change, as would the ecological status of the river.

## 2. Material and Methods

### 2.1. Study Area

**Figure 1:**
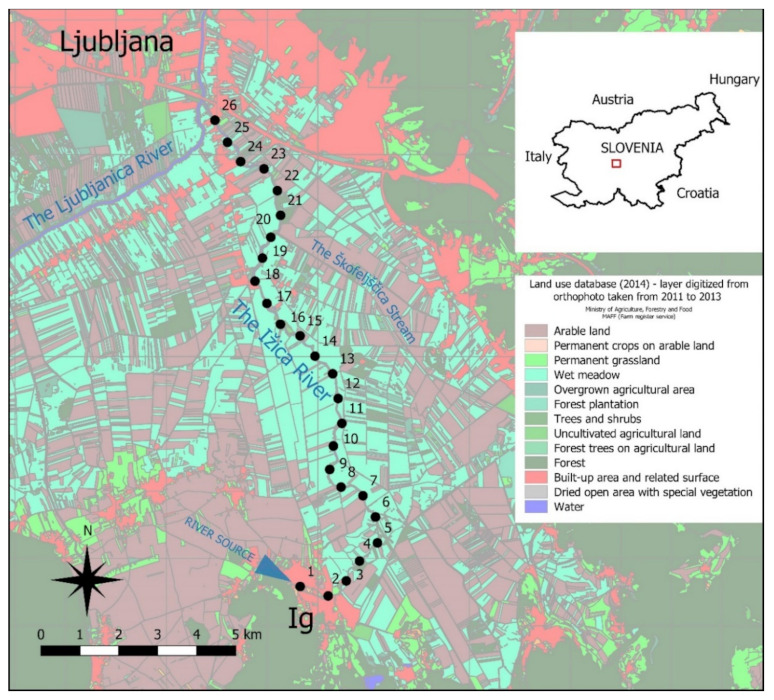
Map of Slovenia with the position of the study area and map of the Ižica River on the Ljubljana Moor. Points represent the starting or ending point of stretches.

The Ižica River is one of the shortest Slovenian rivers, running through the Ljubljana Moor—a 163-km^2^ area of former peatland in the central part of Slovenia (Figure 1) that has been subjected to severe melioration measures in the past. The area lies in a tectonic depression between the Alpine and Dinaric regions, built by alluvial and lacustrine sediments, which are up to 200 m thick [34]. The Alpine region stretches further from Slovenia to Austria and North Italy (Central Europe), whereas the Dinarides is a region which continues further southeast to Croatia, Bosnia and Herzegovina, and Montenegro (Southeastern Europe). Until the end of the 18th century, the area of Ljubljana Moor consisted of a combination of bogs and fens. However, extensive melioration processes in the 19th century changed the area into a mosaic of birch groves, fields, meadows and ditches.

River Ižica has a karst spring characterized by relatively high fluctuations of water discharges. The river’s source is in the center of the town of Ig, and it then flows north through an agricultural landscape, joining the Ljubljanica River after 10.5 km. The river has a vast catchment area on the karstic Dinaric plateau, south of the Moor, which is hard to delimit due to underground flows. It is a slow flowing river with predominantly fine-grained sediments and a low longitudinal profile and low erosion potential [34,35]. Its floodplain has been very dynamic over the past millennia due to the major transformation of the landscape. In 2008, the area was protected in the frame of the 135 km^2^ Ljubljana Moor landscape park. The entire area of this park belongs to the Natura 2000 site, which is protected by European Commission law. 

### 2.2. Macrophyte Survey

Surveys were carried out along the whole stream course in 1996, 2000, and 2016. Macrophytes were surveyed at end of July and in August, the peak vegetation period. They were collected from the water with hooks from a small boat. Using GPS, the river was divided into 26 stretches of length 390 ± 10 m. Macrophyte species abundance was estimated as a relative plant biomass using the five-degree scale: 1 = very rare; 2 = rare; 3 = common; 4 = frequent; 5 = abundant or predominant [36]. This approach is widely used in European countries and is a methodology within the WFD [22]. For further data elaboration, these values were transformed to relative plant abundance using a third power function [37]. The classification of the macrophyte species into the functional groups was done according to Janauer et al. [5].

### 2.3. Assessment of Environmental Conditions

Basic physical and chemical parameters of the water, such as pH, oxygen saturation, oxygen concentration, conductivity and temperature, were measured in the upper (1–8), middle (9–17) and lower (18–26) course of the river with a portable multiprobe (PCD-650, Eutech Singapore). These parameters were investigated simultaneously as macrophyte surveys were performed, and on other dates during the vegetation period. In addition, water samples for analysis of nitrates were collected and analyzed in the laboratory. Water samples were collected from the superficial layers 10–15 cm, which was the same depth as for measurements with the multiprobe. Samples were cooled and filtered through the 0.45-μm glass-fiber filters. The level of NO_3_-N was determined spectrophotometrically using HACH Lange tests (see Table 1).

In 2016, we also assessed ecomorphological parameters such as riparian vegetation structure, land use, structure of the riverbed. The ecomorphological conditions of the river were assessed in all 26 stretches of the Ižica River using the Riparian, Channel, and Environmental (RCE) inventory proposed by Petersen [38] and modified by Germ et al. [39]. We assessed 12 environmental parameters that define land use beyond the riparian zone, the structure of the riparian zone (width, completeness, and type of vegetation) and stream channel morphology (bank structure, bank undercutting, flow dynamics, the bottom type, the presence of detritus, retention structures, and sediment accumulation). Each parameter is comprised of four quality gradient categories where 1 indicates good, close to a natural condition, and 4 indicates the most highly modified condition. That is not necessarily the case in the stream channel morphology, where the changes may also occur due to landscape characteristics or the longitudinal character of the river. Later, we related these parameters to species composition and the presence and abundance of macrophytes.

### 2.4. Data Analyses

The relative plant abundance (RPA) was used to calculate the quantitative significance of individual species in the river [36,40]. Based on the presence and abundance of macrophytes, we calculated the River Macrophyte Index (RMI) [41]. It was developed and intercalibrated [42] to assess the ecological status of Slovenian rivers. Macrophyte species were classified into the functional types (see Table 2 for explanation) and their abundances were grouped. Differences in the average proportions of abundances in functional types of aquatic macrophytes over 20 years were tested for significance with Student’s *t*-test in MS Excel. The mentioned *t*-tests were also used for testing the differences in RMI values along the course of the river and over time.

The similarity of the macrophyte community composition in the sections and different years was checked with detrended correspondence analysis (DCA) with the program package Canoco 4.5 [43], which was also used to test the influence of environmental factors on the aforementioned composition. These relations were tested by canonical correspondence analysis (CCA), since the unimodal gradients in the matrix of species data were revealed beforehand with DCA, where the eigenvalue for the first axis was 0.52 [44]. We used forward selection, where 499 permutations were performed to rank the relative importance of the explanatory variables.

## 3. Results

### 3.1. Water Quality Parameters

The pH of the river water was around 8 (Table 1). The average values of electrical conductivity along the stream ranged from 457 to 476 μS cm^–1^ in 1996, 495–542 μS cm^–1^ in 2000, and 410–447 μS cm^–1^ in 2016. The concentrations of NO_3_-N were largely uniform along the course (Table 1). Average values were 1.1 mg L^−1^ in 1996, while they were 1.0 mg L^–1^ and 0.9 mg L^−1^ in 2000 and 2016, respectively.

### 3.2. Species Richness and Abundance of Macrophytes

**Figure 2:**
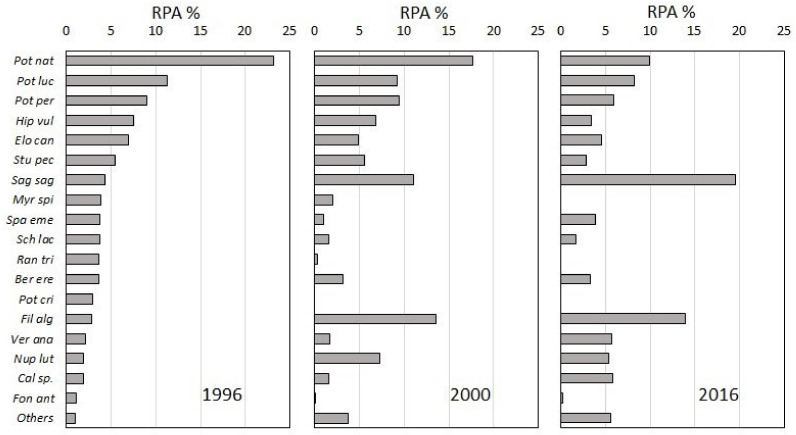
Relative plant abundance (RPA) in 1996, 2000, and 2016. Species with RPA more than 1% abundance are presented. The graphs are based on macrophyte species and abundance in 26 river stretches surveyed in each year. See caption of Table 2 for abbreviations.

**Figure 3:**
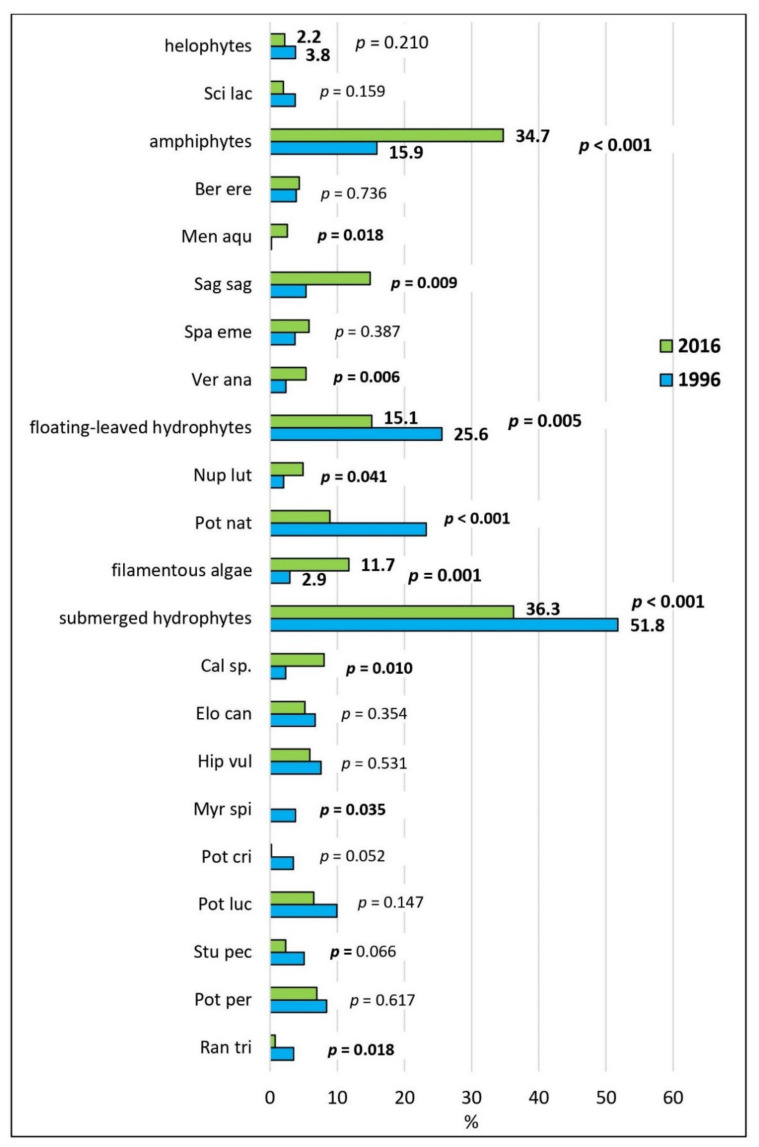
Average proportions of the abundances (in %) of the functional types of aquatic macrophytes, as well as single species with average abundance ≥ 2%. The statistical significance between the years 1996 and 2016 was confirmed with paired *t*-tests. Significant differences over time (*p* < 0.05) are in bold. See caption of Table 2 for abbreviations.

Twenty-seven macrophyte taxa were found in the river (Table 2). However, the recorded number of macrophytes has not changed much over the 20 years, from 24 in 1996, 24 in 2000, to 23 in 2016. On the contrary, the relative plant abundance (RPA) of the most abundant species varies strongly over the sampling years (Figure 2). *Potamogeton natans*, filamentous algae, *P. perfoliatus*, *Sagittaria sagittifolia,* and *P. lucens* reached the highest RPA values. The abundance of *Hippuris vulgaris* was decreasing with time, while the abundance of *S. sagittifolia* was increasing. In 1996 and 2000, *P. natans* was the most abundant species, but in 2016, *S. sagittifolia* dominated. The abundance of *Elodea canadensis* had been slightly decreasing during the studied period. The abundance of the species *Myriophyllum spicatum* decreased between 1996 and 2000, and it could not even be detected in the third survey in 2016. On the other hand, *P. nodosus* was recorded only in 2016. Many species that were newly detected in 2016 exhibit amphibious characteristics (Figure 2) and have significantly increased their proportions (e.g., *S. sagittifolia*, *Veronica anagallis-aquatica*), while the proportion of submerged hydrophytes decreased (Figure 3).

### 3.3. Changes of Macrophyte Assemblages

**Figure 4:**
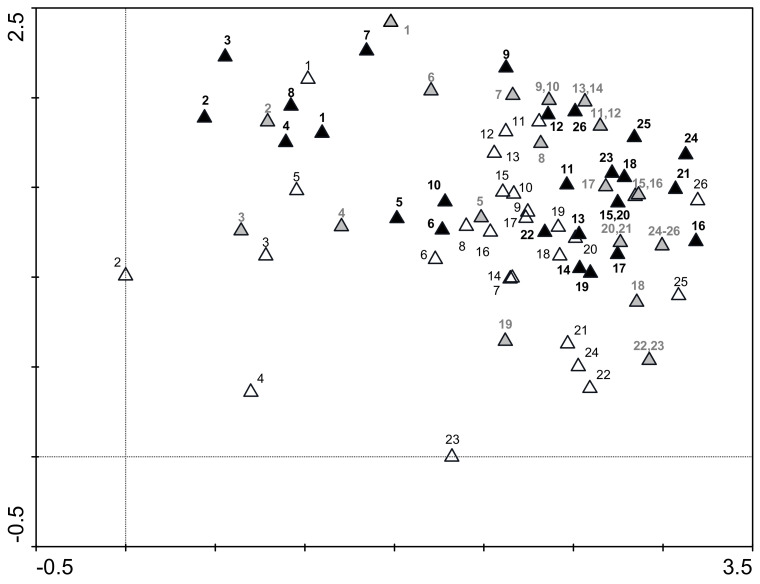
Detrended correspondence analysis ordination diagram showing the similarity among macrophyte assemblages of surveyed stretches in years 1996 (**white triangles**), 2000 (**grey triangles**), and 2016 (**black triangles**). Numbers from 1 to 26 indicate the stretch number (regular, black—1996; bold, grey—2000; bold, black—2016).

The DCA analysis shows the similarity of stretches in terms of the composition of macrophyte assemblages at the peak of the vegetation period in 1996, 2000, and 2016 (Figure 4). The closer the two stretches are on the ordination plot, the more similar the assemblages. In the year 1996, the stretches were more dispersed, but in the subsequent years, the stretches were becoming more uniform.

The same stretches from the lower half of the Ižica River are close together, and thus we recorded similar macrophyte assemblages in different studied years in this part of the river. Stretches from the upper part of the river are more dispersed, indicating greater differences in macrophyte assemblages during the years. The numbers indicating different stretches on the DCA plot increase from left to right, and indicate the longitudinal effect of the river or the gradual downstream change in the assemblage of macrophyte taxa. Given the presence and abundance of macrophyte taxa, the most different sections in all three years are those in the uppermost flow; the middle and lower course stretches are more grouped.

### 3.4. Relationships between Species and Environmental Factors

**Figure 5:**
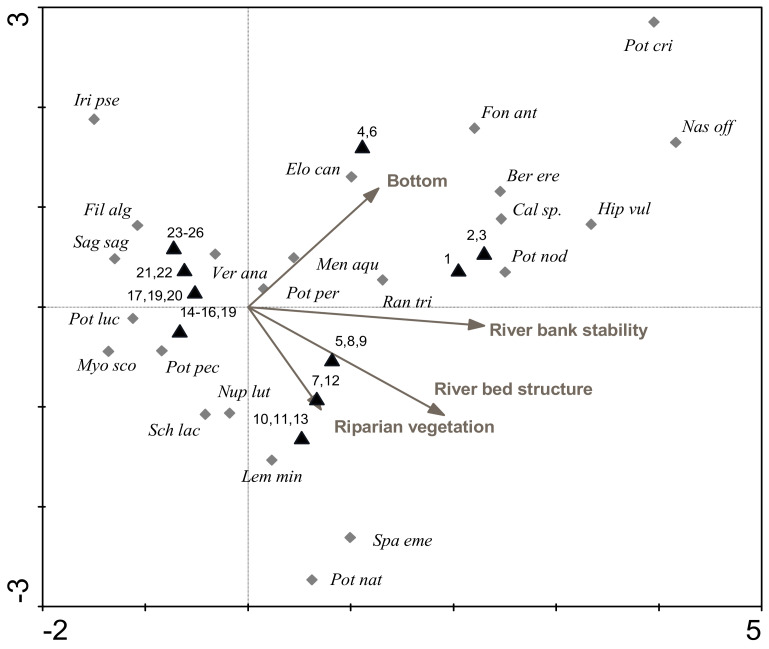
Canonical correspondence analysis (CCA) ordination plot showing the relationship between different locations, macrophytes presence and abundance, and environmental parameters. Abbreviations: *Ber ere—Berula erecta, Cal sp.—Callitriche* spp., *Elo can—Elodea canadensis*, *Fil alg—filamentous algae*, *Fon ant—Fontinalis antipyretica*, *Gly flu—Glyceria fluitans*, *Hip vul—Hippuris vulgaris*, *Iri pse—Iris pseudacorus*, *Lem min—Lemna minor*, *Men aqu—Mentha aquatica*, *Myo sco—Myosotis scorpioides*, *Myr spi—Myriophyllum spicatum*, *Nas off—Nasturtium officinale*, *Nup lut—Nuphar luteum*, *Pot cri—Potamogeton crispus*, *Pot luc—P. lucens*, *Pot nat—P. natans*, *Pot nod—P. nodosus*, *Pot per—P. perfoliatus*, *Stu pec—Stuckenia pectinata*, *Ran tri—Ranunculus trichophyllus*, *Sag sag—Sagittaria sagittifolia*, *Sch lac—Schoenoplectus lacustris*, *Spa eme—Sparganium emersum*, *Ver ana—Veronica anagallis-aquatica*.

In the year 2016, river stretches were assessed using the RCE inventory of the ecomorphological properties of the river ecosystem. Figure 5 shows the similarity among environmental conditions of surveyed stretches. In general, parameters change along the course of the river, but these changes were not linear as is evident from the distribution of the stretches (Figure 5), which form three clusters and are therefore not evenly distributed. Ecomorphological conditions differed more in the upper course of the river.

An ordination plot showing the relationship among the composition of macrophyte community and river morphology parameters revealed that four out of twelve parameters were significant, and together explain 43% of species variability. The most influential parameter was riverbank stability, which explained 20% (*p* = 0.001) of the variability of macrophyte species composition, the riverbed structure explained 10% (*p* = 0.001), vegetation of riparian zone 7% (*p* = 0.001), and type of the bottom explained an additional 5% (*p* = 0.013). The riverbank stability and the riverbed structure are the parameters which correlate most with the first axis and species are clearly distributed along these gradients. Vectors representing the type of the riparian vegetation are most related to the second axis. The stretches of the upstream half of the river show a gradient along the first axis, while the lower part shows the distribution along the second axis and thus the relationship with the vector vegetation of the riparian zone (Figure 5).

### 3.5. Ecological Status

**Figure 6:**
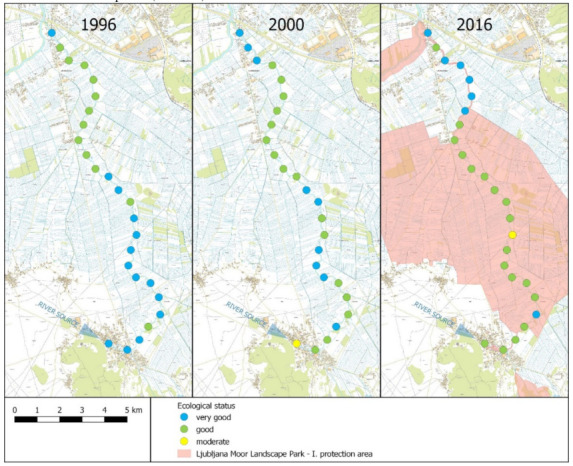
Map of the Ižica River from different years displaying spatial distribution of stretches with different ecological status.

The values of RMI calculated based on macrophyte species showed changes in the ecological status of different sections of the river (Table 3) and in the ecological status of specific stretches along the course, as well as over the years (Figure 6, Table 4). In 1996, more than half of the river stretches (14) showed very good status, while the rest (12) showed good status, with the better status in the upper half of the flow. The situation had already changed in 2000, as the condition of the source changed to moderate, and the nearby stretches changed to good ecological status (Figure 6, Table 4). In 2000, we classified nine stretches as having very good ecological status, sixteen stretches as good, and one stretch had a moderate ecological status. In 2016, we found only seven stretches that were classified to very good ecological status, eighteen to good ecological status and one to moderate ecological status. The location of the stretches with better ecological status was the opposite as in the year 1996, since in 2016, stretches with very good ecological status were concentrated in the lowest part of the river, which had significantly better status than other parts (Table 3).

## 4. Discussion

Luxuriant macrophyte growth and species diversity in all three studied years were supported by favorable conditions, which included sufficient light, type of substratum, and non-torrential water regime of the river [45]. Low water velocity and fine sediment favored the growth of aquatic vegetation, especially in the river’s lower part, as was also shown in other studies [46,47]. The number of macrophyte taxa in the Ižica River was 24 in 1996, 25 in 2000, and 23 in 2016. High RPA values reached taxa such as *P. natans*, *S. sagittifolia* and *P. lucens*, which were most abundant in all the studied years in the lower part of the Ižica River. Both sediment and plant assemblages were more heterogeneous in the upper section.

We found taxa including *R. trichophyllus*, *Berula erecta*, and *Callitriche* spp., indicating low nutrient levels, as well as species indicating high nutrient levels such as *P. natans* [48], which is ecologically the most tolerant species of all pondweeds [49] with respect to eutrophic conditions or turbid water, and is a typical representative in low current velocity waterbodies. Preston [49] reports that *P. natans* thrives in a variety of ecological conditions, from oligotrophic to eutrophic water with different types of substrates. Due to its floating leaves, *P. natans* reduces light penetration into the water column [50,51] and outcompetes other species. *P. natans* often grows in the company of the species *P. lucens* and *P. nodosus* [52], which were also found in the Ižica River in all three study years. *P. nodosus* and *P. natans* usually thrive in similar ecological conditions, but *P. nodosus* prefers a stony substrate [49], thus it was commonly present in the middle flow of the river. On the other hand, *P. lucens* is often the dominant macrophyte in slow flowing rivers with a fine silty substrate. It is often found in diverse assemblages cohabitating with the species *P. natans*, *N. luteum*, *Sparganium erectum*, *Stuckenia pectinata*, and *P. crispus* [53]. Except the species *P. crispus*, these species were found on a muddy substrate in the lower part of the river Ižica. *P. crispus* can also thrive in parts of the river with a faster flow [54], and it is presumed that this is the main reason that it is found in the upper part, where the flow velocity is higher. *B. erecta* was common along the Ižica River, especially in the upper part. It often occurs in alkaline waters in oligotrophic and mesotrophic states [55]. It could be found as an emergent form in shallow water, with leaves partly floating on the surface in slow streams, and as a submerged form [56]. *S. sagittifolia* and filamentous algae prevailed in the lower part of the river in 2016, in the stretches, where in 1996 and 2000, *P. natans* was dominant.

The invasive alien species *Elodea canadensis* has not increased its abundance in the 20-year period (Figure 3). In fact, its relative abundance even decreased, as this species was the fifth most common species in the year 1996, but in only the ninth place 20 years later (Figure 2). This was previously shown by Kuhar et al. [33]. As reasons for low invasiveness of alien aquatic species, Troia et al. [57] report the low nutrient concentration in the water and a diverse macrophyte community, with efficient competitors. In general, the macrophyte assemblage showed a decrease in abundance of species with a wider ecological range, such as *S. pectinata* (*p* = 0.066) and *M. spicatum* (*p* = 0.035), and an increase in the abundance of taxa with narrower ecological range, such as *Callitriche* sp. (*p* = 0.01) and *S. sagittifolia* (*p* = 0.009), the latter as species with amphibious character (Figure 3). Genus *Callitriche* occurred with a high abundance in the upper part of the lowland Ljubljanica River, into which the Ižica River flows [18]. A relatively dry summer and the consequently lower water levels and higher insolation in 2016 [58] may be the reasons for the higher abundance of the amphibious plants, such as *S. sagittifolia*, *Veronica anagallis-aquatica* and *M. aquatica*, compared to other growth forms (Figure 3).

A DCA ordination plot (Figure 4) shows that macrophyte assemblages are changing with time. A longitudinal effect of the downstream change of the macrophyte community is evident. In contrast to stretches of the upper Ižica River, stretches in the lower half of the river were grouped together, showing that, in all years, the macrophyte assemblages of these stretches were similar. This means that the macrophyte community is more homogeneous in the lower part.

Ecomorphological factors with the greatest influence on the species composition of macrophyte community in the river Ižica in 2016 were the vegetation type of the riparian zone, riverbed structure, and the bottom type, that together explained 43% of the species variability. A small number of taxa located in the middle of the ordination plot (Figure 5) correspond to mean values of significant environmental parameters. Hrivnak et al. [47] report that macrophyte community composition in Slovak streams is affected by sediment type, riparian vegetation due to shade of woody vegetation, water depth, NO_2_ level and pH. Halabowski and Lewin [20] showed that conductivity, altitude, land use adjacent to the rivers, and the proportion of sand were the most important factors that affected the distribution of macrophytes in rivers in southern Poland. However, Lewin and Szoszkiewicz [13] showed that non-nutrient parameters play an important role in determining macrophyte presence even in rivers with a relatively high input of nutrients.

The river ecological status over three years was estimated using RMI, which is based on the composition of the macrophyte community and considered a list of taxa indicating different ecological status [41]. The comparison of RMI values along the flow in 1996, 2000, and 2016 showed pronounced differences (Figure 6, Table 3). These values were significantly higher (*p* = 0.004) in 1996 (average = 0.80) than in 2016 (average = 0.72) (Table 4). In 1996, the entire river showed a good to very good ecological status, with the worst status in its lowest course (Table 3 and Table 4). In 2016, the conditions were significantly worse than in 1996 in the upper course (*p* = 0.001), in the middle course (*p* = 0.003), as well as in the entire river (*p* = 0.004) (Table 4). The stretches with very good ecological status were found mainly in the lower course of the river (Figure 6). This was possibly a consequence of the increasing population of Ig, where the river originates. The population development index in the town of Ig between 2002 and 2012 had been above 125, which is the highest within the Ljubljana metropolitan region [59]. The population of the Municipality of Ig in 1991 was 4498, and in 2015 was 7135, which is a 59% increase [60]. The growth of the settlement was not supported with an adequate municipal sewage collecting system, which has affected the structure of the macrophyte community and the ecological status. Better ecological status in 2016 in the lower part of the river (*p* = 0.01) was possibly the result of the improved infrastructure and introduction of more sustainable agricultural practices in the nearby area, as well as along the Škofeljščica stream (Figure 1), which inflows exactly where the Ižica River leaves the most rigorous area of protection within the Landscape Park and Natura 2000 site. The inflow of the cleaner water improved the ecological status of the Ižica River downstream (Figure 6). The settlements on the eastern bank of the Ižica River were connected to the central wastewater treatment plant (WWTP) of Ljubljana in 2014, so there was no such negative impact on the Ižica River system from the area eastward of the river in 2016.

## 5. Conclusions

Favorable ecomorphological conditions and moderate concentrations of nutrients in the river support high plant diversity, and we found a high number of macrophyte taxa in the short river Ižica. The ecological status of the river deteriorated significantly (*p* = 0.004) from 1996 to 2016, particularly in the upper part of the river. The most probable reasons for those changes are the karst character of the river source and its catchment area, respectively, and population growth of the town of Ig around the source of the Ižica River, which was not supported with adequate infrastructure for wastewater treatment. Such long-term studies indicate changes in the environment and human attitude to it, and thus present a basis for future management in the watershed of this and similar watercourses.

## Figures and Tables

**Table 1:** Average values and standard deviations of selected abiotic parameters measured in different stretches of the Ižica River.

Year		pH	Conductivity (μS cm^−1^)	NO_3_-N (mg L^−1^)
1996	average	8.1	466	1.1
	S.D.	0.05	12	0.1
2000	average	7.9	509	1.0
	S.D.	0.2	26	0.2
2016	average	7.8	426	0.9
	S.D.	0.4	17	0.2

**Table 2:** List of aquatic macrophyte taxa recorded in the Ižica River with their codes/abbreviations and functional types (HE—helophytes, AM—amphiphytes, FLH—floating-leaved hydrophytes, FIL—filamentous algae, SM—submerged hydrophytes).

Taxon Name	Code Name	Functional Type
*Berula erecta*	*Ber ere*	HE
*Callitriche* spp.	*Cal sp*	SM
*Elodea canadensis*	*Elo can*	SM
*filamentous algae*	*Fil alg*	FIL
*Fontinalis antipyretica*	*Fon ant*	SM
*Glyceria fluitans*	*Gly flu*	HE
*Hippuris vulgaris*	*Hip vul*	AM
*Iris pseudacorus*	*Iri pse*	HE
*Lemna minor*	*Lem min*	FLH
*Mentha aquatica*	*Men aqu*	AM
*Myosotis scorpioides*	*Myo sco*	AM
*Myriophyllum spicatum*	*Myr spi*	SM
*Nasturtium officinale*	*Nas off*	HE
*Nuphar luteum*	*Nup lut*	FLH
*Potamogeton crispus*	*Pot cri*	SM
*Potamogeton lucens*	*Pot luc*	SM
*Potamogeton natans*	*Pot nat*	FLH
*Potamogeton nodosus*	*Pot nod*	FLH
*Potamogeton perfoliatus*	*Pot per*	SM
*Stuckenia pectinata*	*Stu pec*	SM
*Ranunculus trichophyllus*	*Ran tri*	SM
*Ranunculus circinatus*	*Ran cir*	SM
*Rumex hydrolapathum*	*Rum hyd*	HE
*Sagittaria sagittifolia*	*Sag sag*	AM
*Schoenoplectus lacustris*	*Sch lac*	HE
*Sparganium emersum*	*Spa eme*	AM
*Veronica anagallis-aquatica*	*Ver ana*	AM

**Table 3:** Average values of RMI in the upper, middle and lower course in different years of the survey, and results of testing for significance (*t*-tests) of these changes along the course of the Ižica River. Significant differences (*p* < 0.05) are in bold.

Year	Average RMI, Upper Section:	*p*	Average RMI, Middle Section:	*p*	Average RMI, Lower Section:	Changes of RMI along the Course:
1996	0.836	0.725 n.s.	0.825	**0.030**	0.742	worst status in lower course
2000	0.74	0.653 n.s.	0.75	0.894 n.s.	0.76	no significant changes
2016	0.71	0.070 n.s.	0.65	**0.0003**	0.78	best status in lower course
three years	0.762	0.492 n.s.	0.745	0.439 n.s.	0.762	no significant changes

**Table 4:** Average values of RMI for the entire course of the Ižica River, and for its upper, middle, or lower course in different years of the survey, and results of testing for significance (paired *t*-tests) of these changes over time. Significant differences (*p* < 0.05) are in bold.

year 1996	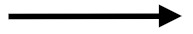	year 2000	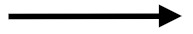	year 2016
average RMI: entire course = 0.80	***p* = 0.018**	average RMI: entire course = 0.75	*p* = 0.151	average RMI: entire course = 0.72
	***p* = 0.004** 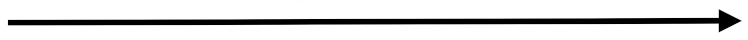			
upper = 0.836		***p* = 0.001**		upper = 0.71
middle = 0.825		***p* = 0.003**		middle = 0.65
lower = 0.742		*p* = 0.127		lower = 0.78

## Data Availability

Data could be available on reasonable request and consent of the first and the last author.
